# EssC is a specificity determinant for *Staphylococcus aureus* type VII secretion

**DOI:** 10.1099/mic.0.000650

**Published:** 2018-04-05

**Authors:** Franziska Jäger, Holger Kneuper, Tracy Palmer

**Affiliations:** Division of Molecular Microbiology, School of Life Sciences, University of Dundee, Dundee, UK

**Keywords:** *Staphylococcus aureus*, protein secretion, Type VII secretion, substrate recognition

## Abstract

The type VII protein secretion system (T7SS) is found in actinobacteria and firmicutes, and plays important roles in virulence and interbacterial competition. A membrane-bound ATPase protein, EssC in *Staphylococcus aureus*, lies at the heart of the secretion machinery. The EssC protein from *S. aureus* strains can be grouped into four variants (EssC1–EssC4) that display sequence variability in the C-terminal region. Here we show that the EssC2, EssC3 and EssC4 variants can be produced in a strain deleted for *essC1*, and that they are able to mediate secretion of EsxA, an essential component of the secretion apparatus. They are, however, unable to support secretion of the substrate protein EsxC, which is only encoded in *essC1*-specific strains. This finding indicates that EssC is a specificity determinant for T7 protein secretion. Our results support a model in which the C-terminal domain of EssC interacts with substrate proteins, whereas EsxA interacts elsewhere.

The type VII secretion system (T7SS) is found primarily in bacteria of the actinobacteria and firmicutes phyla and secretes proteins that lack cleavable N-terminal signal peptides. The system is best characterized in mycobacteria, where it is designated ESX, and pathogenic members of the genus can encode up to five copies of the secretion machinery [1, 2]. Substrates of the T7SS may vary in size, but are usually α-helical in nature. Every T7SS analysed to date secretes at least one protein of the WXG100 superfamily. Proteins of this family are small helical hairpins that have a conserved WXG amino acid motif in a short loop between the two helices [3, 4]. A non-cleaved sequence located close to the C-termini of some WXG100 proteins acts as a signal for T7 secretion [5–8]. Some studies have suggested that the WXG motif may act alongside the C-terminal region as a bipartite targeting signal [9, 10]. WXG100 proteins are secreted as folded dimers; in actinobacteria these are heterodimers of paired WXG100 proteins, whereas in firmicutes they may also be homodimers [9]. The T7SS also secretes much larger substrates that share a similar four-helical bundle arrangement of the WXG100 protein dimers [10–12]. Some T7 substrates interact with chaperones prior to secretion and there is evidence that secretion of LXG domain substrates in firmicutes is dependent on complex formation with a WXG100 protein partner [13–15].

There are commonalities and differences between the T7SS of actinobacteria and firmicutes [16]. A membrane-embedded ATPase of the FtsK/SpoIIIE family termed EccC/EssC is found in all T7SSs. In both systems the protein shares a similar overall topology, with two transmembrane domains that are usually followed by three P-loop ATPase domains at the C-terminus. Although all three P-loop ATPase domains are capable of binding ATP, mutagenesis studies have indicated that only ATP hydrolysis by domain 1 is essential for T7 secretion [8, 17]. In actinobacteria, a hexameric arrangement of the EccC ATPase lies at the centre of a 1.8MDa complex that also contains six copies of the EccB, EssD and EccE proteins [18]. In firmicutes, homologues of EccB, D and E are absent and a distinct set of membrane proteins, EsaA, EssA and EssB, work alongside the ATPase, EssC, to mediate T7 secretion [19–23]. In *Staphylococcus aureus* and *Bacillus subtilis* a secreted WXG protein, EsxA, and a small cytoplasmic protein, EsaB, are also required for T7SS activity [19, 20, 22–24] (Fig. 1a).

**Fig. 1.**
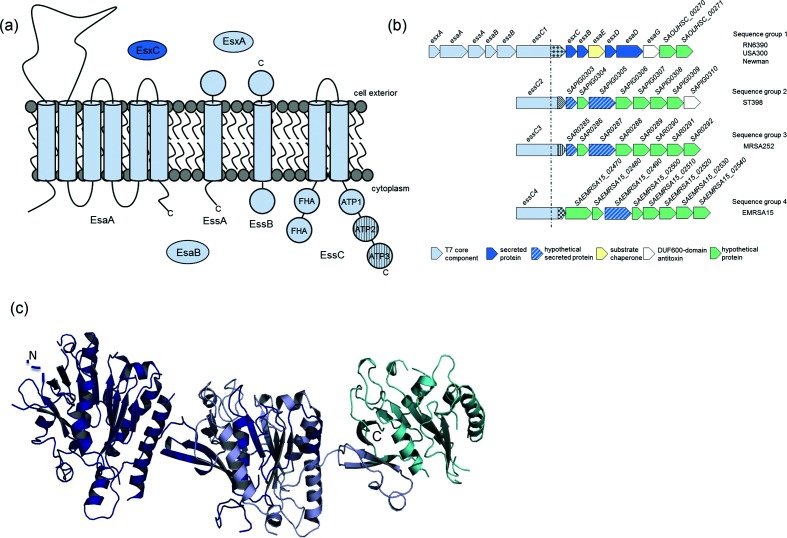

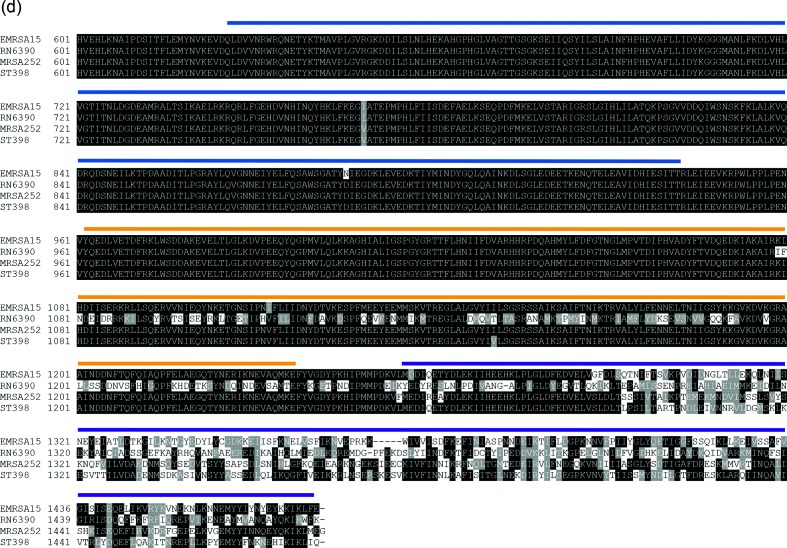
Sequence variability in *S. aureus* EssC. (a) The *S. aureus* T7 secretion machinery. Components that are essential for T7 secretion are shown in light blue with their subcellular locations. The hatched domains of EssC indicate sequence-variable regions. The substrate protein EsxC, found only in strains with the EssC1 variant, is shown in dark blue. (b) Genetic organization of the *S. aureus ess* locus in the four different *ess* strain variants. Since the 3′ boundaries of the *ess* loci are not known, the first eight genes downstream of *essC* are shown in each case. The dotted line indicates the approximate position of *essC* sequence divergence and the shading at the 3′ end of *essC* represents the region of sequence variability. (c) Structural model of the ATPase domains of *S. aureus* EssC (generated using amino acids 601–1078 of EMRSA15 EssC) using Phyre2 (www.sbg.bio.ic.ac.uk/~phyre/) with the structure of EccC from *Thermomonospora curvata* [8] as a template. Dark blue shading, residues 601–1078, very highly conserved; light blue shading, residues 1079–1289 (where the EssC1 sequence diverges from the remaining EssC); cyan shading, residues 1290–1479 (variable C-terminal region). (d) Alignment of EssC sequences from the indicated *S. aureus* strains. The alignment was generated using clustal W (www.ch.embnet.org/software/ClustalW.html) and shaded using Boxshade (https://embnet.vital-it.ch/software/BOX_form.html), and is shown from amino acid 600 onwards. The blue, yellow and purple lines above the alignment delimit the extent of ATPase domains 1, 2 and 3, respectively, based on the Phyre model generated in (c).

The EccC/EssC ATPase has previously been implicated in substrate recognition. It was shown that the C-terminal domain of EccCb1 interacted with the EsxB substrate [5, 25], while the EccC ATPase domains have been co-crystallized with a peptide from the EsxB C-terminus [8]. Crosslinking and co-purification experiments have identified complexes of *S. aureus* EssC with substrates EsaD (also called EssD) and EsxC [14, 26]. Further evidence in support of a role for EssC in substrate recognition comes from genomic analysis of *S. aureus* [27]. It was noted that there was sequence variability at the *ess* locus across different *S. aureus* strains. Genes coding for the core components EsxA-EssB are highly conserved (Fig. 1b), as is the 5′ end of *essC*, but the 3′ portion of the gene falls into one of four sequence groupings [27]. The *essC* sequence type strictly co-varies with the sequence of adjacent 3′ genes, some of which are known or strongly predicted to encode secreted substrates. This would be consistent with the C-terminal variable region of EssC playing a role in substrate recognition. In this study we have addressed this hypothesis directly by assessing whether EssC proteins from the EssC2, EssC3 and EssC4 classes can support the secretion of the EssC1 substrate, EsxC [28], and of the core component, EsxA.

*S. aureus* EssC proteins are approximately 1480 amino acids in length and have a common domain organization, with two forkhead-associated (FHA) domains at their N-termini, followed by two transmembrane domains, and three repeats of a P-loop ATPase domain at their C-termini ([29, 30]; Fig. 1a). Sequence analysis indicates that *S. aureus* EssC proteins are almost sequence invariant until part way through the second ATPase domain, where the EssC1 variant, found in strains such as RN6390, Newman and USA300, starts to diverge (Fig. 1c, d). The EssC2, EssC3 and EssC4 variants are more similar to one another, and share almost identical sequences until ATPase domain 3, where they also start to vary (Fig. 1c, d). Of the four ATPases, variants 2 (from strain ST398) and 3 (from strain MRSA252) are the most similar (Fig. 1d).

We have previously constructed an in-frame deletion of *essC* in strain RN6390 and shown that this results in the inability to export both the core machinery component, EsxA, and the substrates EsxC and EsaD [14, 20]. This secretion deficiency could be rectified by the reintroduction of EssC1 encoded on plasmid pRMC2 [31]. Fig. 2(a) shows that production of EssC1 could be also restored when it was encoded on the expression vector pRAB11 [32], and that the reintroduction of plasmid-encoded EssC1 resulted in strong secretion of both EsxA and EsxC in the RN6390 Δ*essC* strain.

**Fig. 2.**
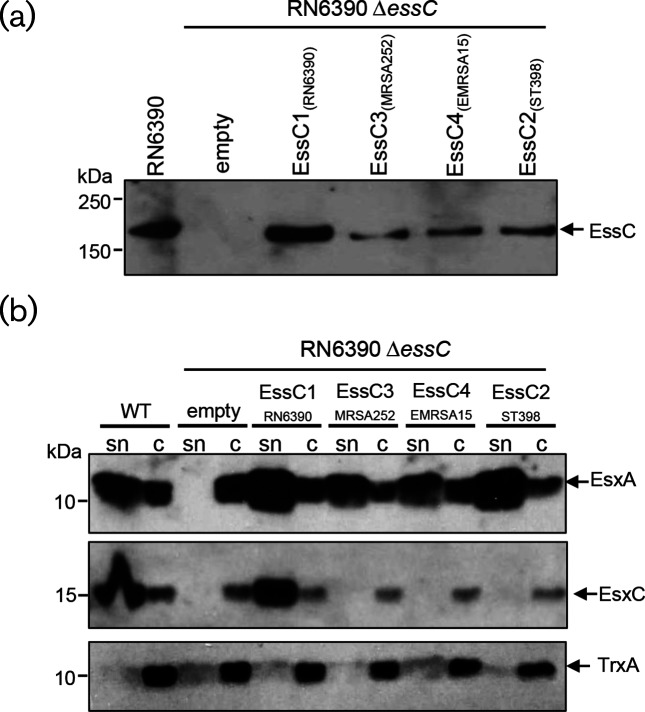
Non-cognate EssC variants support secretion of EsxA but not EsxC. (a, b) Strain RN6390 or the isogenic *essC* deletion strain carrying pRAB11 (empty) or pRAB11 encoding the indicated *essC* variant was subcultured into TSB medium supplemented with 1 µM haemin [34] and either 25 ng ml^−1^ (RN6390 Δ*essC*/pEssC_RN6390_) or 100 ng ml^−1^ (RN6390 Δ*essC*/pEssC_MRSA252_/pEssC_ST398_/pEssC_EMRSA15_) anhydrotetracycline (ATC) to induce plasmid-encoded gene expression. The strains were grown aerobically until an OD_600_ of 2 was reached, after which (a) 10 µl of OD_600_ 1 adjusted cells were separated on an 8 % bis-Tris acrylamide gel and analysed by Western blotting using anti-EssC antisera [20], or (b) the cultures were separated into supernatant and whole-cell fractions and the equivalent of 200 µl of culture supernatant (sn) and 10 µl of resuspended cell sample adjusted to an OD_600_=1 were separated on a 15 % bis-Tris gel and immunoblotted using the antiserum raised against EsxA [20], EsxC [20] or the cytosolic control, TrxA [35].

Next, we amplified the genes for *essC2* (from strain ST398), *essC3* (from strain MRSA252) and *essC4* (from strain EMRSA15), and also cloned these into pRAB11 (see Table S1 for the oligonucleotides used for these experiments, available in the online version of this article). We first confirmed that the three variant EssC proteins could be stably produced in the RN6390 Δ*essC* strain background. To this end, anhydrotetracycline (ATC) was added to induce plasmid-encoded production of EssC and whole-cell samples were analysed by blotting with an EssC antiserum. It should be noted that the antiserum used was raised against a truncated protein covering the last two ATPase domains of the EssC1 variant [20]. As shown in Fig. 2(a), each of the EssC2, EssC3 and EssC4 variants could be recognized by this antibody, but not so strongly as the cognate EssC1, probably due to a lack of conservation of epitopes in this region of the protein. We conclude that all EssC variants can be produced in strain RN6390.

Next, we asked whether the variant EssC proteins in RN6390 could support T7 protein secretion. Fig. 2(b) (top panel) shows that secretion of the EsxA core component was indeed supported by each of these EssC proteins, indicating that each EssC variant was functional in the heterologous strain background. However, none of the EssC variants were able to support secretion of the substrate protein, EsxC. Taken together, these results confirm that EssC is a specificity determinant for substrate secretion by the *S. aureus* T7SS. The findings strongly suggest that the sequence-invariant regions of EssC proteins are involved in mediating interactions with the conserved T7 core components, including the secreted protein EsxA (which has >99 % sequence identity across all sequenced *S. aureus* strains), and that the sequence-variable region is involved in substrate recognition. This might imply that EsxA and EsxC are secreted by different mechanisms.

Finally, it is interesting to note that the secretion of all known substrates mediated by the EssC1 variant is dependent on a chaperone protein, EsaE/EssE [14, 26]. Some substrates of the actinobacterial T7SS also interact with specific chaperones of the EspG family to ensure delivery to the cognate secretion machinery [13, 33], although other substrates appear to be exported independently of a specific chaperone [2]. No protein with any detectable sequence homology to either EsaE or EspG is encoded at the *ess* loci of the *essC2*, *essC3* or *essC4* strain variants. In future it will be interesting to determine whether the mechanism of substrate targeting differs across the Ess subtypes in *S. aureus*.

## Supplementary Data

58 Supplementary File 1

## References

[1] Gröschel MI, Sayes F, Simeone R, Majlessi L, Brosch R (2016). ESX secretion systems: mycobacterial evolution to counter host immunity. Nat Rev Microbiol.

[2] Ates LS, Houben EN, Bitter W (2016). Type VII Secretion: a highly versatile secretion system. Microbiol Spectr.

[3] Renshaw PS, Lightbody KL, Veverka V, Muskett FW, Kelly G (2005). Structure and function of the complex formed by the tuberculosis virulence factors CFP-10 and ESAT-6. EMBO J.

[4] Sundaramoorthy R, Fyfe PK, Hunter WN (2008). Structure of *Staphylococcus aureus* EsxA suggests a contribution to virulence by action as a transport chaperone and/or adaptor protein. J Mol Biol.

[5] Champion PA, Stanley SA, Champion MM, Brown EJ, Cox JS (2006). C-terminal signal sequence promotes virulence factor secretion in *Mycobacterium tuberculosis*. Science.

[6] Daleke MH, Ummels R, Bawono P, Heringa J, Vandenbroucke-Grauls CM (2012). General secretion signal for the mycobacterial type VII secretion pathway. Proc Natl Acad Sci USA.

[7] Poulsen C, Panjikar S, Holton SJ, Wilmanns M, Song YH (2014). WXG100 protein superfamily consists of three subfamilies and exhibits an α-helical C-terminal conserved residue pattern. PLoS One.

[8] Rosenberg OS, Dovala D, Li X, Connolly L, Bendebury A (2015). Substrates control multimerization and activation of the multi-domain ATPase motor of type VII secretion. Cell.

[9] Sysoeva TA, Zepeda-Rivera MA, Huppert LA, Burton BM (2014). Dimer recognition and secretion by the ESX secretion system in *Bacillus subtilis*. Proc Natl Acad Sci USA.

[10] Solomonson M, Setiaputra D, Makepeace KA, Lameignere E, Petrotchenko EV (2015). Structure of EspB from the ESX-1 type VII secretion system and insights into its export mechanism. Structure.

[11] Ekiert DC, Cox JS (2014). Structure of a PE-PPE-EspG complex from *Mycobacterium tuberculosis* reveals molecular specificity of ESX protein secretion. Proc Natl Acad Sci USA.

[12] Korotkova N, Freire D, Phan TH, Ummels R, Creekmore CC (2014). Structure of the *Mycobacterium tuberculosis* type VII secretion system chaperone EspG5 in complex with PE25-PPE41 dimer. Mol Microbiol.

[13] Daleke MH, van der Woude AD, Parret AH, Ummels R, de Groot AM (2012). Specific chaperones for the type VII protein secretion pathway. J Biol Chem.

[14] Cao Z, Casabona MG, Kneuper H, Chalmers JD, Palmer T (2016). The type VII secretion system of *Staphylococcus aureus* secretes a nuclease toxin that targets competitor bacteria. Nat Microbiol.

[15] Whitney JC, Peterson SB, Kim J, Pazos M, Verster AJ (2017). A broadly distributed toxin family mediates contact-dependent antagonism between gram-positive bacteria. Elife.

[16] Unnikrishnan M, Constantinidou C, Palmer T, Pallen MJ (2017). The enigmatic Esx proteins: looking beyond mycobacteria. Trends Microbiol.

[17] Ramsdell TL, Huppert LA, Sysoeva TA, Fortune SM, Burton BM (2015). Linked domain architectures allow for specialization of function in the FtsK/SpoIIIE ATPases of ESX secretion systems. J Mol Biol.

[18] Beckham KS, Ciccarelli L, Bunduc CM, Mertens HD, Ummels R (2017). Structure of the mycobacterial ESX-5 type VII secretion system membrane complex by single-particle analysis. Nat Microbiol.

[19] Burts ML, Williams WA, Debord K, Missiakas DM (2005). EsxA and EsxB are secreted by an ESAT-6-like system that is required for the pathogenesis of *Staphylococcus aureus* infections. Proc Natl Acad Sci USA.

[20] Kneuper H, Cao ZP, Twomey KB, Zoltner M, Jäger F (2014). Heterogeneity in *ess* transcriptional organization and variable contribution of the Ess/Type VII protein secretion system to virulence across closely related *Staphylocccus aureus* strains. Mol Microbiol.

[21] Mielich-Süss B, Wagner RM, Mietrach N, Hertlein T, Marincola G (2017). Flotillin scaffold activity contributes to type VII secretion system assembly in *Staphylococcus aureus*. PLoS Pathog.

[22] Baptista C, Barreto HC, São-José C (2013). High levels of DegU-P activate an Esat-6-like secretion system in *Bacillus subtilis*. PLoS One.

[23] Huppert LA, Ramsdell TL, Chase MR, Sarracino DA, Fortune SM (2014). The ESX system in *Bacillus subtilis* mediates protein secretion. PLoS One.

[24] Casabona MG, Buchanan G, Zoltner M, Harkins CP, Holden MTG (2017). Functional analysis of the EsaB component of the *Staphylococcus aureus* Type VII secretion system. Microbiology.

[25] Stanley SA, Raghavan S, Hwang WW, Cox JS (2003). Acute infection and macrophage subversion by *Mycobacterium tuberculosis* require a specialized secretion system. Proc Natl Acad Sci USA.

[26] Anderson M, Ohr RJ, Aly KA, Nocadello S, Kim HK (2017). EssE promotes *Staphylococcus aureus* ESS-dependent protein secretion to modify host immune responses during infection. J Bacteriol.

[27] Warne B, Harkins CP, Harris SR, Vatsiou A, Stanley-Wall N (2016). The Ess/Type VII secretion system of *Staphylococcus aureus* shows unexpected genetic diversity. BMC Genomics.

[28] Burts ML, Dedent AC, Missiakas DM (2008). EsaC substrate for the ESAT-6 secretion pathway and its role in persistent infections of *Staphylococcus aureus*. Mol Microbiol.

[29] Tanaka Y, Kuroda M, Yasutake Y, Yao M, Tsumoto K (2007). Crystal structure analysis reveals a novel forkhead-associated domain of ESAT-6 secretion system C protein in *Staphylococcus aureus*. Proteins.

[30] Zoltner M, Ng WM, Money JJ, Fyfe PK, Kneuper H (2016). EssC: domain structures inform on the elusive translocation channel in the Type VII secretion system. Biochem J.

[31] Jäger F, Zoltner M, Kneuper H, Hunter WN, Palmer T (2016). Membrane interactions and self-association of components of the Ess/Type VII secretion system of *Staphylococcus aureus*. FEBS Lett.

[32] Helle L, Kull M, Mayer S, Marincola G, Zelder ME (2011). Vectors for improved Tet repressor-dependent gradual gene induction or silencing in *Staphylococcus aureus*. Microbiology.

[33] Phan TH, Ummels R, Bitter W, Houben EN (2017). Identification of a substrate domain that determines system specificity in mycobacterial type VII secretion systems. Sci Rep.

[34] Casabona MG, Kneuper H, Alferes de Lima D, Harkins CP, Zoltner M (2017). Haem-iron plays a key role in the regulation of the Ess/type VII secretion system of *Staphylococcus aureus* RN6390. Microbiology.

[35] Miller M, Donat S, Rakette S, Stehle T, Kouwen TR (2010). *Staphylococcal* PknB as the first prokaryotic representative of the proline-directed kinases. PLoS One.

