# Supplementary material for: EssC is a specificity determinant for Staphylococcus aureus type VII secretion
Source: Microbiology (Reading). 2018 Apr 5;164(5):816–20. doi: 10.1099/mic.0.000650 (PMC5994694; doi:10.1099/mic.0.000650)
Supplement: Supplementary File 1 [file mic-164-816-s001.pdf]

Name	Sequence (5' – 3')	Restriction site
essCRN6390fw	AAATAGATCTAGGACTGAGGCAAG	*Bgl*II
essCRN6390rev	CCTATTGAATTCATTGCTATTTAAACC	*Eco*RI
essCfwdSacI	AAAGAGCTCTAGGACTGAGGCAAAGACAATGC	*Sac*I
essCr2EMRSA15	CAAATCTCATAGAGCTCTCGTTTTATTCAAATAA	*Sac*I
essCr2ST398	CATAATTGAGCTCCCTATTGAATTAATTTTATTTT	*Sac*I
essCrevMRSA252	CTTTATGAGCTCTATCCCTCCATTAG	*Sac*I

Table S1. Oligonucleotides used in this study. The *essC* gene from RN6390 was amplified using oligonucleotides essCRN6390fw and essCRN6390rev and cloned into plasmid pRAB11 as a *Bgl*II-*Eco*RI fragment. The other three essC genes were amplified using the same forward primer (essCfwdSacI) and a strain-specific reverse primer and cloned into pRAB11 as *Sac*I fragments. All inserts were confirmed for directionality and fidelity by DNA sequencing.
